# A Digital Simulation Model of Broadband Phased Array RF System and Its Application

**DOI:** 10.3390/s25134133

**Published:** 2025-07-02

**Authors:** Jia Ding, Huaizong Shao, Jianxing Lv, Fake Ding

**Affiliations:** 1 School of Information and Communication Engineering, University of Electronic Science and Technology of China, Chengdu 611731, China; dingji15@uestc.edu.cn; 2 Nanhu Laboratory, Defined RF Chip and System Research Center, Jiaxing 314001, China; 15852707697@163.com (J.L.); 15757368626@163.com (F.D.)

**Keywords:** phased array system, behavior-level simulation model, simulation software, delay control scheme

## Abstract

The design and application of broadband phased array RF links is a complex and highly precise endeavor. To achieve optimal performance, it is essential to compare and validate multiple schemes during the system design phase. Utilizing simulation models to simulate system structures and validate parameters can effectively reduce research and development time and costs. This article takes the broadband phased array RF system (RFS04) currently being developed by Nanhu Laboratory as a reference and constructs a behavioral-level signal simulation model. Through this model, the antenna pattern of RFS04 was generated, and the relationship between beam pointing accuracy and delay quantization bit number was analyzed. The 3 dB beam coverage range of the 18 GHz antenna array was calculated, and the synthesis scheme of multi-phased arrays was explored. Additionally, the correspondence between the angle measurement accuracy and signal-to-noise ratio of the RFS04 system was analyzed. This article also measured the delay module parameters of the RF system and developed a correction strategy for the delay control scheme. Through simulation calculations and laboratory testing, it has been proven that this strategy can effectively improve delay accuracy. After applying the modified delay control scheme to the RFS04 simulation model, the beam pointing accuracy during phased array antenna scanning was significantly enhanced. The model research and integrated simulation software construction of the broadband phased array RF system provide an efficient and accurate simulation tool for system design and optimization.

## 1. Introduction

**Figure 1:**
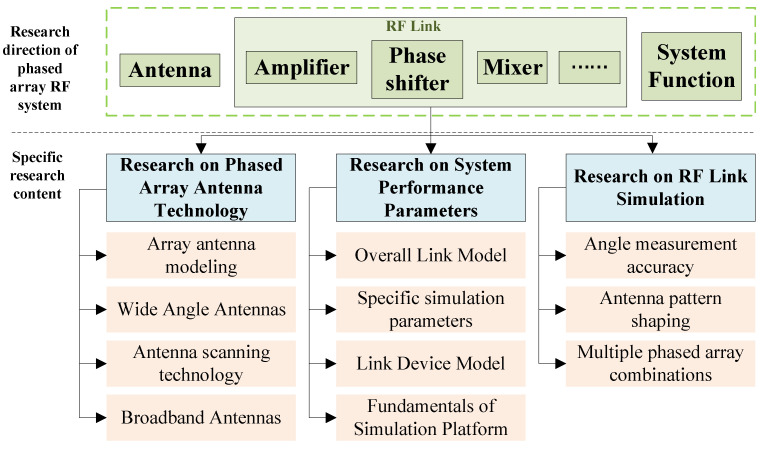
Main research content of phased array RF system.

A phased array system is an advanced electronic system that employs multiple array element antennas for signal transmission and reception. By precisely adjusting the signal delay between these array elements, the system can achieve electronic beam scanning without the need for mechanical moving parts. This distinctive capability bestows phased array systems with rapid response times and exceptional reliability, thereby rendering them highly advantageous across a diverse array of applications. Phased array technology originated in the late 1930s and has undergone several generations of iterative development, evolving from passive phased arrays to active phased arrays, and eventually to digital phased arrays [1]. Today, phased array systems have been widely adopted in both military and civilian fields, including aerospace, geographic mapping, meteorological detection, and medical imaging. Looking to the future, the primary development trends of phased array technology are expected to focus on the universalization, miniaturization, and broadband expansion of arrays [2]. Currently, the research on phased array RF systems primarily concentrates on three key areas: the development of phased array antenna technology, the simulation and modeling of RF links, and the investigation of system performance parameters (such as beam scanning and angle measurement accuracy). The main research directions of phased array RF systems are illustrated in Figure 1.

As shown in Figure 1, phased array systems mainly include three parts: an antenna [3], an RF link [4], and system function [5]. The relevant research mainly focuses on phased array antenna technology, system performance parameters, and RF link simulation.

**Figure 2:**
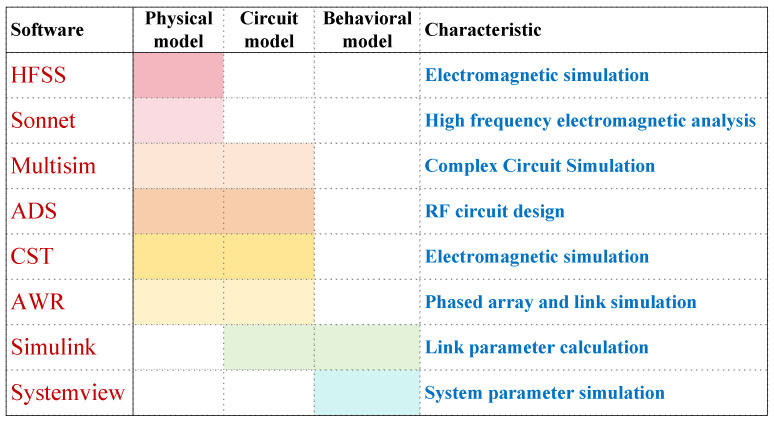
Comparison of characteristics of various simulation software.

With the increasing complexity of the electromagnetic environment in modern battlefields and the emergence of new targets and threats, countries are accelerating the systematic development of the electromagnetic spectrum, promoting the broadband and reconfigurable technology of RF systems, and deepening equipment applications to maintain strategic advantages in future electromagnetic spectrum operations [6]. In this context, our unit actively conducts research on universal definable components and ultra-wideband channels of RF systems based on independently developed RF chips. However, the design of broadband phased array RF systems is highly complex and precise [7]. In order to meet the system performance and development cycle requirements, it is crucial to compare multiple schemes, evaluate parameters, and verify them in the system design phase [8]. Using simulation technology to simulate the system link structure and verify parameters [9,10] is an effective approach to achieve this goal, which can significantly reduce the time and capital costs of system development. The simulation platform used in the relevant model research has the following main characteristics [11,12], as shown in Figure 2.

From the perspective of simulation methods, the existing RF link simulation technologies are mainly divided into three categories: electromagnetic parameter simulation, circuit parameter simulation [13], and behavioral simulation of link components [14,15]. Electromagnetic parameter simulation and circuit parameter simulation can provide relatively accurate results, but modeling is complex and computationally intensive, making it difficult to comprehensively model a complete RF system [16]. Behavioral-level simulation models do not focus on specific device structures but only simulate their response characteristics, which can achieve rapid analysis of link parameters [17]. However, the current behavioral-level model simulation platforms have limitations [18]: they cannot cover phased array antennas and all links in RF systems [19]; on the other hand, focusing solely on hardware circuit parameters [20] makes it difficult to directly evaluate the functional indicators of the system, such as beam scanning coverage and angle measurement accuracy [21,22].

To address the aforementioned challenges, this study relies on the RF module independently developed by Nanhu Laboratory and its measured parameters to construct a digital model library of the RF module. Based on this, simulation software tailored for the design of broadband phased array RF systems was developed [23]. This software provides simulation tools for designing new broadband phased array RF systems [24], supports multi-scheme comparison, and integrates with system parameter calculation [25], link optimization, and problem analysis [26].

**Figure 3:**
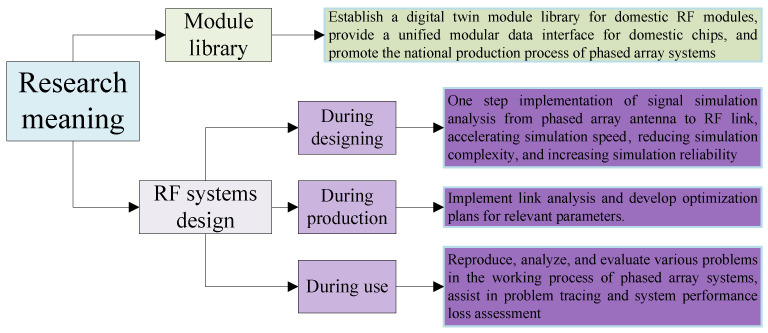
Main research significance.

As shown in Figure 3, the research on phased array simulation systems is of great value in model library establishment and RF system design, containing three stages: during designing, during production, and during use. It provides a fast, accurate, and efficient signal simulation tool for the design optimization and analysis of phased array RF systems, which is highly significant for promoting the rapid development of future electronic information systems.

## 2. Broadband Phased Array RF System and Its Simulation Model

In recent years, phased array technology has continued to advance, and its application scenarios are also expanding. Modeling and simulation work is essential to meet the design requirements of various phased array systems. For phased array RF systems, the primary research focuses on the two core components: phased array antennas and RF links.

### 2.1. Phased Array Radio Frequency System

Based on the broadband phased array RF system under development at Nanhu Laboratory, the composition was studied and analyzed. Based on the existing system architecture, a universal simulation software for broadband phased array RF systems has been developed, providing an efficient simulation tool for the design of the system.

#### 2.1.1. System Structure

**Figure 4:**
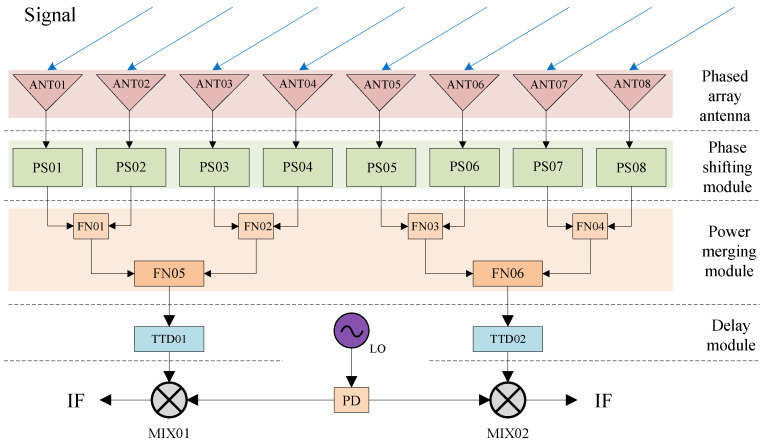
Basic structure of broadband phased array RF system.

As depicted in Figure 4, the fundamental structure of a broadband phased array RF system (Nanhu Laboratory, Jiaxing, China) primarily consists of phased array antennas, delay modules, power combining modules, mixing modules, and so on. In addition to these key functional components, each module comprises electronic components like amplifiers, attenuators, equalizers, and so forth. The electromagnetic signal in the target direction is initially received by the antenna and converted into a voltage signal. After undergoing amplification, phase shifting, delay, and other processing, the signal is down-converted to the effective frequency range by a mixer, while being mixed with thermal noise and stray noise from the device, and ultimately outputs an intermediate frequency signal. The intermediate frequency signal is sampled and digitized by an ADC, then enters the digital link. Phased array scanning is achieved by adjusting the signal phase of each array element antenna receiving link via phase shifters and delays.

#### 2.1.2. Simulation Model Architecture

**Figure 5:**
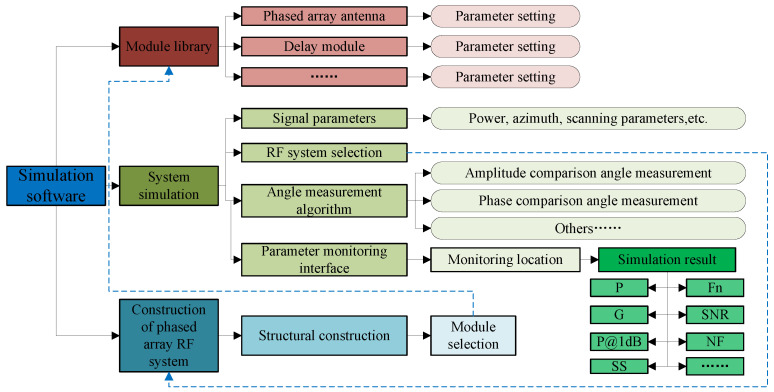
Basic structure of simulation software.

Based on the fundamental structure of phased array RF systems, simulation models for each module were developed, and specialized simulation software was designed accordingly. As shown in Figure 5, the simulation software primarily consists of three components: module library establishment, system model construction, and system simulation.

Module library establishment: Based on simulation models and laboratory-measured parameters, digital twin models of each module are constructed, including phased array antennas, phase-shifting modules, delay modules, and so on. System model building: Design the basic composition structure of the phased array RF system, and select models from the module library for each part. System simulation: It includes three parameter-setting pages: the signal and scanning parameter setting interface, the RF system selection interface, and the angle measurement method selection interface.

Through this simulation software, the module library can be used to quickly build the required broadband phased array RF system. It can not only output circuit parameters such as gain, noise figure, and antenna pattern of the RF part but also directly obtain the angle measurement accuracy of the system.

**Figure 6:**
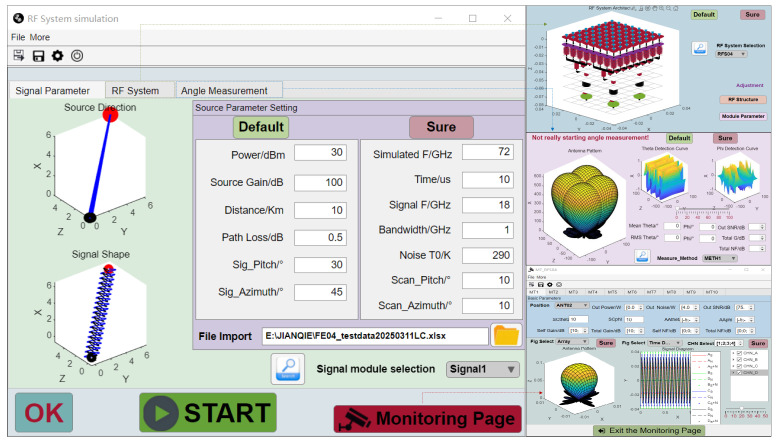
Display interface of simulation software.

The interface of the broadband phased array RF system simulation software (Version 1.0, hereinafter referred to as RFSS) is depicted in Figure 6. On the left is the interface for setting signal and scanning parameters. Among the three images on the right, the top two are interfaces for configuring other simulation parameters, while the lower right image is the node parameter monitoring interface. This interface allows users to view the output results of different modules within the simulation system.

Through the UI panel, the parameters of each module (delay module, phase-shift module, power merging module, frequency conversion module, sampling module, etc.) can be configured and transmitted back to the simulation interface. By clicking the STAR button, the calculation of all link parameters and system parameters can be achieved.

### 2.2. Phased Array Antenna

A phased array antenna serves as the initial module in broadband RF systems. The target’s electromagnetic signal enters the RF system via the phased array antenna and propagates as a voltage signal within the system. A phased array antenna is a key module in RF systems, and its directional pattern is a critical indicator for evaluating system performance. In simulations, the arrangement of antenna elements can be customized, and the simulation software can perform synthesis operations on their directional patterns. Typically, phased array antennas are arranged in one-dimensional or two-dimensional configurations.

#### 2.2.1. One-Dimensional Phased Array Antenna

**Figure 7:**
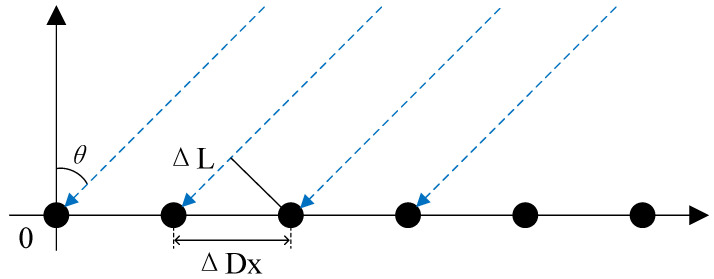
One-dimensional phased array antenna.

As depicted in Figure 7, in a one-dimensional phased array antenna, the signal path varies depending on the position of the antenna elements. The path difference between adjacent antenna elements is given by [27]

(1) $$\Delta L=\Delta D_{x}sin\theta ,$$

So, the phase difference between adjacent antenna elements is

(2) $$\Delta \Phi =\frac{\Delta L}{\lambda }2\pi ,$$

For a one-dimensional N-element phased array antenna, if the directional pattern of each element is $F_{n}$, then the overall directional pattern of the phased array antenna is

(3) $$F_{N}=\sum_{n=1}^{N}F_{n}e^{j\Delta \Phi \left(n-1\right)}=F_{n}\frac{1-e^{j\frac{\Delta D_{x}sin\theta }{\lambda }2\pi N}}{1-e^{j\frac{\Delta D_{x}sin\theta }{\lambda }2\pi }},$$

#### 2.2.2. Two-Dimensional Phased Array Antenna

**Figure 8:**
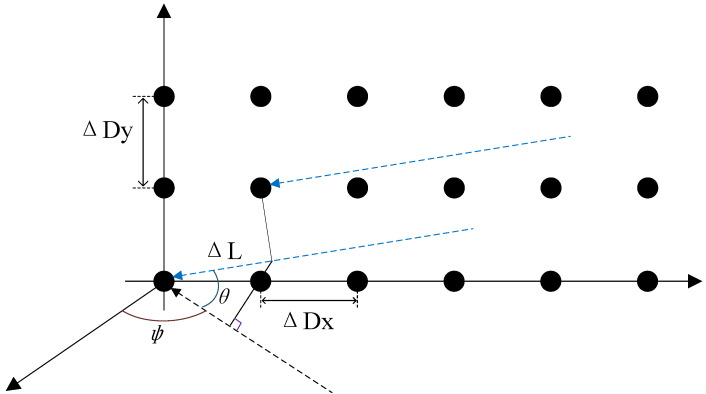
Two-dimensional phased array antenna.

As shown in Figure 8, the elements of the two-dimensional antenna array are positioned in the YOZ plane, also referred to as the YOZ coordinate system. When the signal reaches the antenna elements at different positions, path differences occur. The path difference between adjacent antenna elements is given by [28]

(4) $$\Delta L=\Delta D_{x}cos\theta sin\phi +\Delta D_{y}sin\theta ,$$

For a two-dimensional N-element phased array antenna, if the directional pattern of each element is $F_{n}$, then the overall directional pattern of the phased array antenna is [29]

(5) $$\begin{array}{cc} F_{N} & =\sum_{n=1}^{N}\sum_{m=1}^{M}e^{j\Delta \Phi_{x}\left(n-1\right)}e^{j\Delta \Phi_{y}\left(m-1\right)}\\ \; & =F_{n}\frac{1-e^{j\frac{\Delta D_{x}cos\theta sin\phi }{\lambda }2\pi N}}{1-e^{j\frac{\Delta cos\theta sin\phi }{\lambda }2\pi }}\cdot \frac{1-e^{j\frac{\Delta D_{y}sin\theta }{\lambda }2\pi M}}{1-e^{j\frac{\Delta D_{y}sin\theta }{\lambda }2\pi }}, \end{array}$$

### 2.3. Phased Array RF Link

#### 2.3.1. Phase-Shifting Module and Delay Module

**Figure 9:**
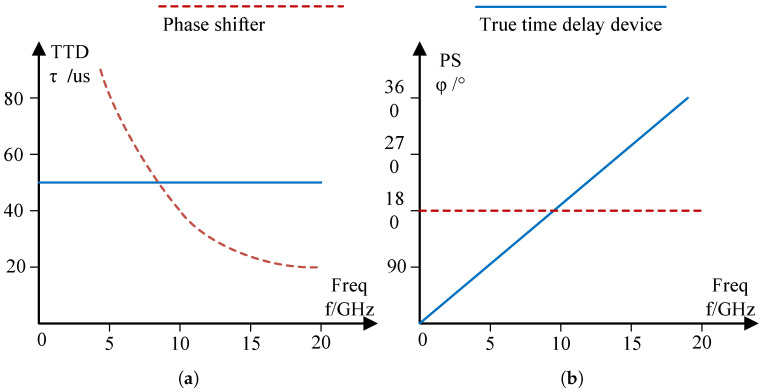
Comparison of phase shifters and delay devices: (**a**) delay parameters; (**b**) phase-shift parameters.

Unlike mechanical scanning antennas, phased array antennas achieve beam scanning by adjusting the path differences of different array elements, without the need for mechanical components. For narrowband phased array systems, path differences are mainly achieved through phase shifters; The broadband phased array system requires the cooperation of delay and phase shifter. For example, the images of the delay- and phase-shift parameters of the 50 μs delay and $180^{\circ }$ phase shifter as a function of frequency are shown in Figure 9.

As shown in Figure 9a, the delay device achieves signal delay through real path differences, so its delay parameters remain unchanged for signals of different frequencies. However, the phase shift of the signal increases with frequency, as shown by the blue lines in Figure 9a,b. Similarly, as shown by the red dashed lines in Figure 9a,b, the phase shifter directly acts on the phase of the signal, so the phase shift does not vary with frequency. But, when passing through a phase shifter with fixed parameters, the delay parameter of the signal is inversely proportional to the frequency.

#### 2.3.2. Series Parallel Module

When the signal passes through electronic components such as amplifiers, the noise of the components themselves will be superimposed on the signal, resulting in a difference between the output signal-to-noise ratio and the input signal-to-noise ratio. In the linear gain region, the noise figure is defined as

(6) $$F=\frac{S_{0}/N_{0}}{S_{1}/N_{1}}=\frac{S_{0}\left(N_{0}+N_{e}\right)G}{S_{0}N_{0}G}=1+\frac{N_{e}}{N_{0}}=1+\frac{kT_{e}B}{kT_{0}B}=1+\frac{T_{e}}{T_{0}},$$

Among them, $S_{0}$ and $N_{0}$ are the input signal and noise power, $S_{1}$ and $N_{1}$ are the output signal and noise power, $N_{e}$ is the equivalent device noise to the input terminal, *G* is the device gain, *k* is the Boltzmann constant, *B* is the channel bandwidth, and $T_{0}$ and $T_{e}$ are the equivalent temperatures corresponding to device noise and input noise, respectively.

**Figure 10:**
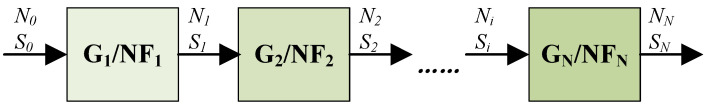
Serial link.

When multiple devices are connected in series, their corresponding images are shown in Figure 10:

If only two devices are considered in series, the total noise figure can be expressed as

(7) $$\begin{array}{cc} F_{0∼2} & =\frac{S_{0}/N_{0}}{S_{2}/N_{2}}=\frac{S_{0}\left(\left(N_{0}+N_{e1}\right)G_{1}+N_{e2}\right)G_{2}}{S_{0}G_{1}G_{2}N_{0}}\\ \; & =\frac{\left(N_{0}+N_{e1}\right)G_{1}}{G_{1}N_{0}}+\frac{N_{e2}}{G_{1}N_{0}}=F_{1}+\frac{F_{2}-1}{G_{1}}, \end{array}$$

Therefore, for *N* series connected devices, the total noise figure is

(8) $$F_{0∼N}=F_{1}+\frac{F_{2}-1}{G_{1}}+\frac{F_{3}-1}{G_{1}G_{2}}+\ldots +\frac{F_{i}-1}{G_{1}G_{2}\ldots G_{i-1}}+\ldots +\frac{F_{N}-1}{G_{1}G_{2}\ldots G_{N-1}},$$

In passive phased array systems, in addition to series links, parallel circuits are also important components.

**Figure 11:**
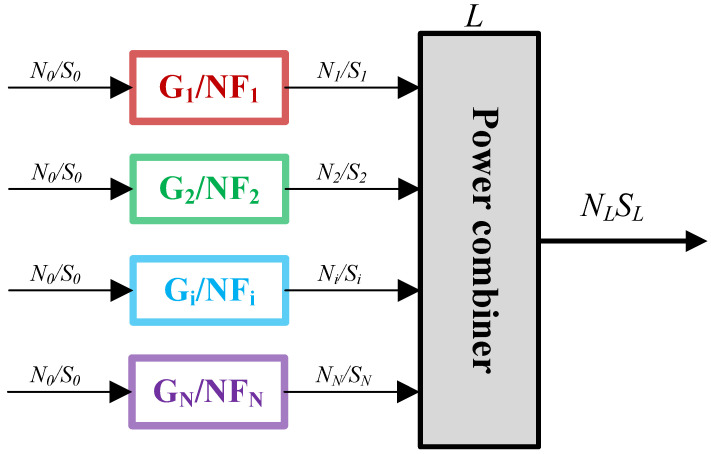
Parallel circuit.

In the parallel link of Figure 11, the signal input from the front-end passes through a section of the link and finally reaches the input of the power combiner. At this point, the signals and noise entering the power combiner are

(9) $$\begin{array}{cc} \; & S_{i}=S_{0}G_{i},\\ \; & N_{i}=\left(N_{0}+N_{e}\right)G_{i}=N_{0}F_{i}, \end{array}$$

In Equation (9), $G_{i}$ and $F_{i}$ are the gain and noise figure of the i-th branch, and $S_{i}$ and $N_{i}$ are the signal and noise transmitted from the i-th branch to the power combiner, respectively. For a power combiner, only 1/N of the signal power from the input port is transmitted to the output port. Output signal and output noise are

(10) $$\begin{array}{cc} \; & S_{out}=\sum_{i=1}^{N}\frac{S_{0}G_{i}}{LN},\\ \; & N_{out}=\sum_{i=1}^{N}\frac{N_{0}G_{i}}{LN}+N_{L}, \end{array}$$

In Equation (10), *L* is the attenuation coefficient of the power combiner, *N* is the number of parallel links, and $N_{L}$ is the output noise of the power combiner itself, $N_{L}=kBT_{eL}/L=kBT_{0}\left(1-1/L\right)$. However, the premise for this equation to hold is that the signal phase entering the power combiner is consistent. When the phase of the input signal is inconsistent [25]

(11) $$\begin{array}{cc} \; & U_{S,out}=\sum_{i=1}^{N}\sqrt{\frac{G_{i}}{LN}}U_{S,in,i},\\ \; & S_{out}=\frac{{\left|U_{S,out}\right|}^{2}}{R},\\ \; & U_{N,out}=\sum_{i=1}^{N}\sqrt{\frac{G_{i}F_{i}}{LN}}U_{N,in,i},\\ \; & N_{out}=\frac{{\left|U_{N,out}\right|}^{2}}{R}, \end{array}$$

In Equation (11), $U_{S,in}$ and $U_{N,in}$ are the input signal and noise voltage, respectively, $U_{S,out}$ and $U_{N,out}$ are the output signal and noise voltage, respectively. So, the total noise figure of the parallel link is [20]

(12) $$\begin{array}{cc} F_{L} & =\frac{S_{in,all}/N_{in,all}}{S_{out}/N_{out}}\\ \; & =\frac{{\left|\sum_{i=1}^{N}\sqrt{\frac{1}{N}}U_{S,in,i}\right|}^{2}{\left|\sum_{i=1}^{N}\sqrt{\frac{G_{i}F_{i}}{LN}}U_{N,in,i}+U_{N,L}\right|}^{2}}{{\left|\sum_{i=1}^{N}\sqrt{\frac{G_{i}}{LN}}U_{S,in,i}\right|}^{2}{\left|\sum_{i=1}^{N}\sqrt{\frac{1}{N}}U_{N,in,i}\right|}^{2}}, \end{array}$$

When the gain of each branch is completely consistent with the noise figure, the above equation can be simplified as

(13) $$F_{L}=F+\frac{L-1}{G},$$

## 3. Simulation Results

Referring to the RFS04 parameters of the broadband phased array RF system being developed by Nanhu Laboratory, the phased array antenna adopts a uniform array arrangement of 8×8, with an element spacing of 0.008 m, and the system supports bandwidth signal reception of 2–18 GHz.

### 3.1. Antenna Pattern and Beam Direction

**Figure 12:**
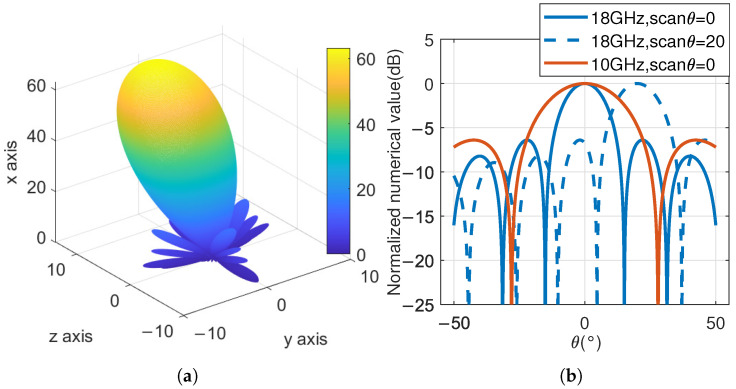
Antenna pattern: (**a**) the three-dimensional image of the antenna pattern when the antenna scanning angle $\theta =10^{\circ }$, $\phi =0^{\circ }$; (**b**) the cross-sectional image of the antenna pattern at position.

From the three-dimensional antenna pattern in Figure 12a, it can be seen that the antenna gain in the beam scanning direction is the highest. In Figure 12b, the solid and dashed blue lines correspond to the cross-sectional images of the 18 GHz antenna at $\theta =10^{\circ }$ and $\phi =0^{\circ }$, respectively. The simulation results are basically consistent with the set scanning parameters. The orange line represents the cross-sectional image of the 10 GHz antenna when $\theta =0^{\circ }$. Compared to the 18 GHz antenna image, the beamwidth is significantly larger, which is consistent with expectations.

The accuracy of beam scanning is a key parameter for determining whether the scanning is in place, and it is also an important factor affecting the overall performance of the system. For narrowband phased array systems, beam scanning is mainly achieved through phase shifters, so the quantization bit number of the phase shifters is a key factor affecting beam pointing.

(14) $$\Delta \theta =\sqrt{\frac{\sum_{i=1}^{N}{\left(\theta_{m,i}-\theta_{0,i}\right)}^{2}}{N}},$$

**Figure 13:**
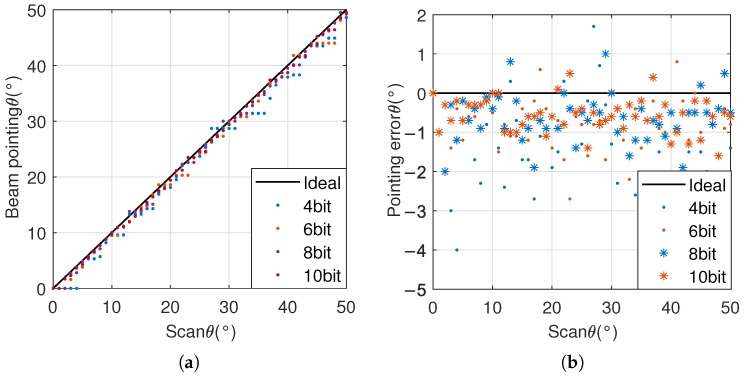
Beam pointing accuracy: (**a**) comparison between actual beam direction and scanning direction; (**b**) beam pointing error.

Calculate the beam pointing error at various angles within the scanning range and obtain the corresponding root mean square error, as shown in Equation (14). Among them, $\theta_{m}$ and $\theta_{0}$ are the measured and theoretical values of beam pointing, respectively. Taking the phased array system as an example, when using phase shifters with different quantization bits, the beam direction of the 18 GHz antenna system is shown in Figure 13.

In Figure 13, the phase-shift range of all phase shifters is $\left[0^{\circ },360^{\circ }\right]$, and the images corresponding to the ideal delay and 4-bit, 6-bit, 8-bit, and 10-bit phase shifters are shown in the figure. During simulation, the azimuth angle ϕ is kept at $0^{\circ }$, and the scanning angle θ gradually increases from $0^{\circ }$ to $50^{\circ }$.

Figure 13a shows the simulation results of actual beam pointing, and Figure 13b shows the error situation of beam pointing. From Figure 13b, it can be seen that, as the number of quantization bits increases, the beam pointing error gradually decreases.

The beam pointing errors of 2 GHz, 10 GHz, and 18 GHz frequencies are shown in Table 1:

From Table 1, it can be seen that, as the number of quantization bits increases, the beam pointing error gradually decreases and the beam pointing accuracy gradually improves, but the magnitude of the accuracy improvement gradually decreases. In practical applications, considering both beam pointing error and device cost, a 6∼8 bit phase shifter is usually selected.

### 3.2. Beam Coverage Area and Multi-Faceted Array Synthesis

In the antenna pattern shown in Figure 12, if the area with normalized gain greater than −3 dB is truncated, it can be referred to as the 3 dB beam coverage area. In an ideal situation, when the phased array is not scanning, both θ and ϕ pointing at the beam are $0^{\circ }$, and the shape of the 3 dB beam coverage area is approximately circular. However, as the scanning angle increases, the shape distortion of the 3 dB beam coverage area will become increasingly severe. In addition, when scanning at a large angle, the main beam efficiency and sidelobe gain of the antenna will also significantly deteriorate. To avoid this situation, the maximum scanning angle should be minimized as much as possible.

For two-dimensional phased array antenna systems, tilting the array is an effective method to reduce the maximum scanning angle. In the YOZ coordinate system, assuming the scanning angle range is $\theta_{1}$∼$\theta_{2}$ and $\phi_{1}$∼$\phi_{2}$, then the calculation formula for the optimal array tilt angle is

(15) $$tan\theta_{T}=-\frac{cos\theta_{1}-cos\theta_{2}}{sin\theta_{1}-sin\theta_{2}}cos\phi_{2},$$

For the phased array RF system under development, when the scanning elevation angle is $\theta =\left[0^{\circ },60^{\circ }\right]$ and the scanning azimuth angle $\phi =\left[-45^{\circ },45^{\circ }\right]$, the optimal array tilt angle $tan\theta_{T}=22.21^{\circ }$ is calculated. Taking the 18 GHz channel as an example, we have developed corresponding beam scanning strategy, which is shown in Table 2.

**Figure 14:**
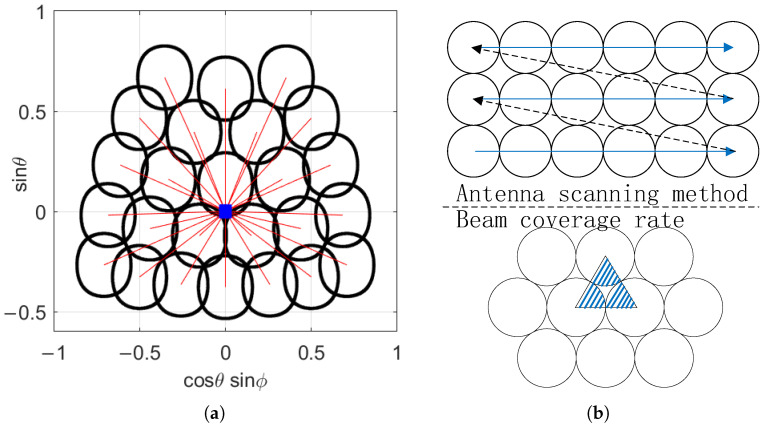
Beam scanning method and coverage area: (**a**) when scanning the antenna according to the angle in Table 2, in the YOZ coordinate system with the antenna as the reference, the blue square represents the position of the phased array antenna, the red line represents the beam direction, and the black line represents the 3 dB beam coverage area; (**b**) the antenna adopts a Z-shaped scanning method, and the arrangement of wave positions refers to the staggered arrangement method.

At this point, the 3 dB beam coverage area corresponding to each beam scanning position is shown in Figure 14a:

(16) $$\eta_{1}=\frac{A_{cover,all}}{A_{aim}},\eta_{2}=\frac{A_{cover,all}}{\sum_{i=1}^{N}A_{cover,i}},$$

In Equation (16), $\eta_{1}$ and $\eta_{2}$ represent the beam coverage and effective utilization of the beam, respectively. Among them, $A_{cover,all}$ is the effective coverage area, $A_{aim}$ is the target area, and $A_{cover,i}$ is the 3 dB beam coverage area corresponding to each scanning position.

For common wave position arrangements, as shown in Figure 14b, the column arrangement has an ideal beam coverage rate of 78.5% and an effective beam utilization rate of 100%. If full coverage is achieved, the effective utilization rate of the beam is only 63.7%. As shown in Figure 14b, under ideal conditions, the staggered arrangement has a beam coverage rate of 90.7% and an effective utilization rate of 100%. If full coverage is achieved, the utilization rate of the beam is only 82.7%. The scanning method used in this article has a beam coverage rate of 91.9% and a beam utilization rate of 68.7%, which is closer to the staggered arrangement and meets the requirement of beam coverage rate of 85%.

**Figure 15:**
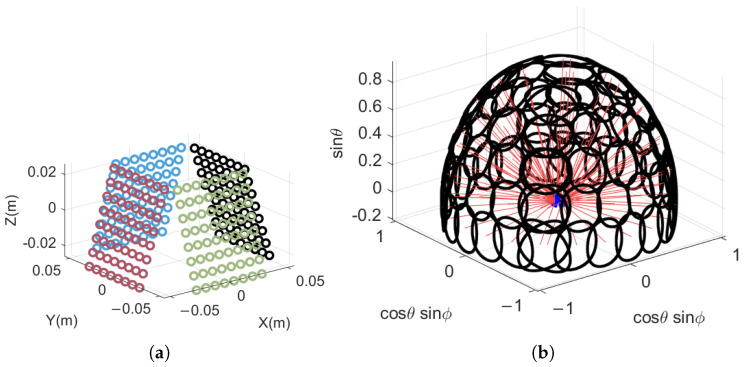
Synthesis of multiple phased arrays: (**a**) element arrangement of synthesized phased array; (**b**) the coverage area of the synthesized 3 dB beam.

The above array and wave position arrangement can meet the target detection within the range of $\phi =\left[-45^{\circ },45^{\circ }\right]$, with an effective azimuth range of $90^{\circ }$. Therefore, to achieve all-round detection, a four-sided phased array system can be used for combination. The combined array layout and 3 dB beam coverage range are shown in Figure 15.

Figure 15 shows a new phased array composed of four arrays and the corresponding beam coverage area in 3D space. Compared with single-sided phased array, the beam coverage and utilization remain unchanged. But, at the edge position $\phi =-135^{\circ }$/$-45^{\circ }$/$45^{\circ }$/$135^{\circ }$ of the array scan, there is a repeated scan. When the repeated scanning is canceled, the beam efficiency of the four-sided array system can still reach about 90%, and the beam utilization rate is about 80%, which is significantly improved compared to the single-sided array.

### 3.3. Angle Measurement Accuracy

Single pulse angle measurement method is a common angle measurement method in phased array systems. Taking the sum–difference amplitude-comparison angle measurement method as an example, we explored the theoretical angle measurement accuracy of phased array systems.

For an 8×8 phased array antenna, it is divided into four 4×4 antenna subarrays: A/B/C/D.

(17) $$\begin{array}{cc} \; & B\left(\theta \right)=\frac{\left|F_{C}\right|+\left|F_{D}\right|-\left|F_{A}\right|-\left|F_{B}\right|}{\left|F_{A}\right|+\left|F_{B}\right|+\left|F_{C}\right|+\left|F_{D}\right|},\\ \; & B\left(\phi \right)=\frac{\left|F_{B}\right|+\left|F_{D}\right|-\left|F_{A}\right|-\left|F_{C}\right|}{\left|F_{A}\right|+\left|F_{B}\right|+\left|F_{C}\right|+\left|F_{D}\right|}, \end{array}$$

Equation (17) is the calculation formula for the sum difference ratio angle measurement, which can generate a ratio lookup table for various orientations. In practical applications, the corresponding target orientation can be obtained through a lookup table based on the actual ratio of received signals.

**Figure 16:**
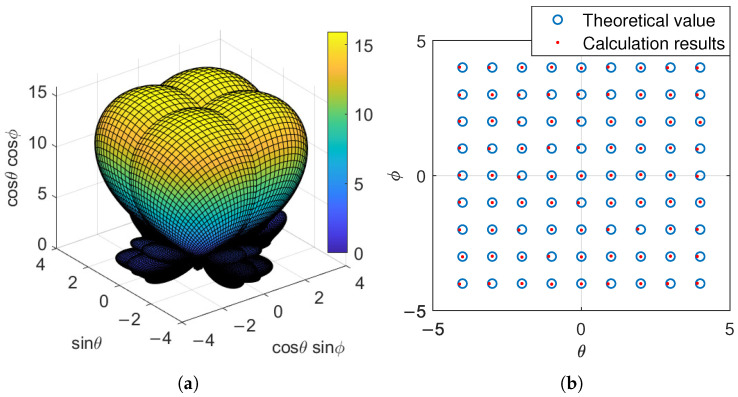
Amplitude measurement method: (**a**) subarray antenna pattern; (**b**) angle measurement accuracy.

When the scanning angle of the phased array is $\left(0^{\circ },0^{\circ }\right)$, the beam directions of the A/B/C/D subarrays are $\left(-5^{\circ },-5^{\circ }\right)$, $\left(-5^{\circ },5^{\circ }\right)$, $\left(5^{\circ },-5^{\circ }\right)$, and $\left(5^{\circ },5^{\circ }\right)$, respectively. The corresponding antenna subarray pattern is shown in Figure 16a, and some results of the angle measurement accuracy are shown in Figure 16b.

Assuming the target direction is $\theta =-6^{\circ }$:$1^{\circ }$:$6^{\circ }$, $\phi =-6^{\circ }$:$1^{\circ }$:$6^{\circ }$. There are a total of 169 simulation directions. The signal frequency is 18 GHz, and 1000 points are uniformly sampled over 10 cycles of the signal. Take the average of the ratios of these 1000 points and determine the target direction based on the lookup table. When the signal-to-noise ratio is 20 dB, the actual target azimuth and measurement results are shown in Figure 16b.

**Figure 17:**
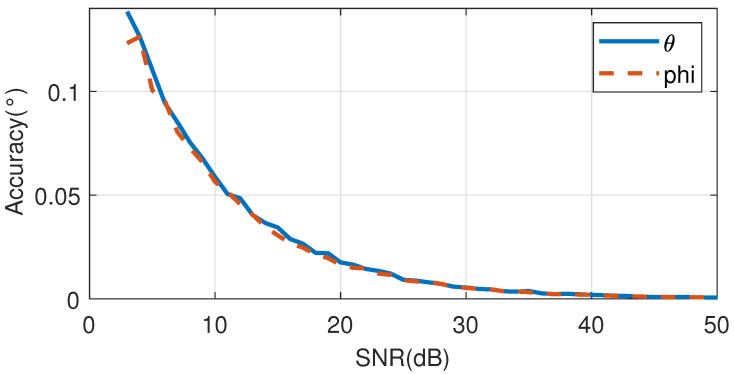
Angle measurement accuracy varies with signal-to-noise ratio image.

From Figure 16b, it can be seen that there is an error between the calculated signal direction and the theoretical value. Statistically analyze the data of all simulation positions to obtain the angle measurement accuracy. When the signal-to-noise ratio changes, the variation in angle measurement accuracy is shown in Figure 17.

In Figure 17, the horizontal axis represents signal-to-noise ratio and the vertical axis represents angle measurement accuracy. As shown in Figure 17, the angle measurement accuracy gradually improves with the increase in signal-to-noise ratio. Therefore, in the design of phased array RF systems, in order to ensure angle measurement accuracy, the signal-to-noise ratio of the RF system output should be maximized.

## 4. Application of the System Model

At present, Nanhu Laboratory has completed the structural design of the phased array RF system, and the design and production of each module have also been completed and are currently undergoing testing.

**Figure 18:**
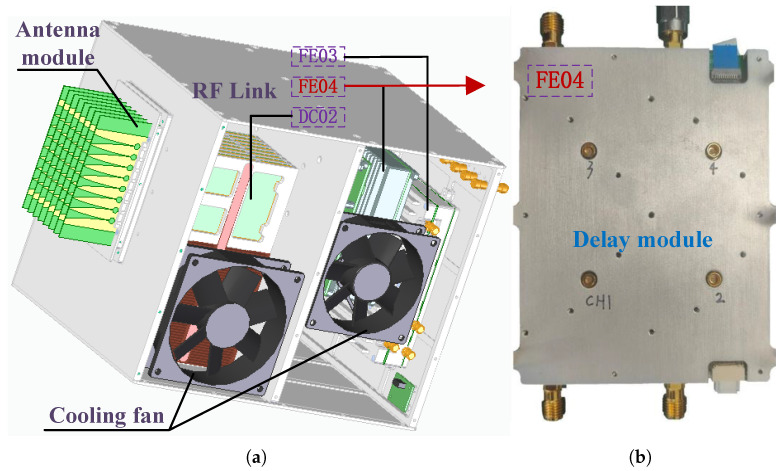
The broadband RF system developed by Nanhu Laboratory: (**a**) schematic diagram of RF system structure; (**b**) delay module of RF system.

As is shown in Figure 18a, the RF system mainly consists of phased array antenna, RF link, and cooling fan. The RF link includes RF modules such as FE03, FE04, DC02, etc. As is shown in Figure 18b, FE04 is a delay function module that includes four link channels. Currently, the delay module FE04 is being tested to verify its basic functional parameters. The basic design parameters of FE04 are shown in Table 3.

### 4.1. Test Results of FE04

**Figure 19:**
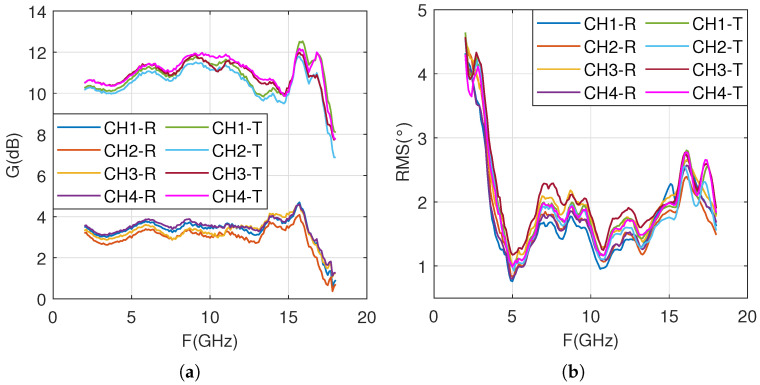
Test results of the delay module: (**a**) transmission and reception gain; (**b**) root mean square error of delay state.

Figure 19 shows the test results of the delay module FE04, where Figure 19a shows the channel gain and Figure 19b shows the delay accuracy. FE04 contains four channels, which are used for receiving and transmitting in two working states, so there are a total of eight parameter curves in the figure.

From Figure 19a, it can be seen that the average receiving gain is about 3.5 dB, and the transmitting gain is about 11 dB. The gain changes of the four channels are basically consistent, and the gain flatness in the mid frequency and low frequency bands is good, meeting the design specifications. However, the gain flatness in the high-frequency range is poor, especially around 17 GHz, where the flatness of the receiving gain is about 2 dB and the flatness of the transmitting gain is at its worst, about 3.5 dB. As shown in Figure 19b, the delay accuracy variation curves of the four channels are basically consistent. The delay accuracy is poor in the low-frequency range, but it meets the design requirement of less than 5 ps.

### 4.2. Delay Correction Result

**Figure 20:**
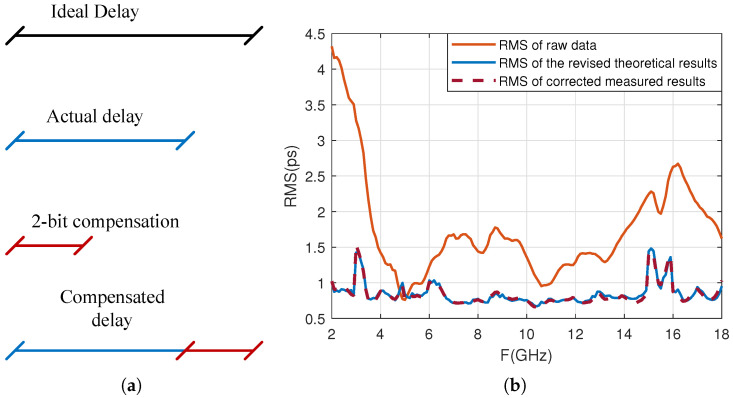
Delay correction method and results: (**a**) schematic diagram of delay compensation method; (**b**) comparison of delay accuracy before and after correction.

The actual hardware circuit of the delay module adopts an 8-bit quantization redundancy design, with a minimum delay and delay step of 2.5 ps. When in use, the lowest two bits are masked, the module employs 6-bit quantization, the minimum delay and delay step are changed to 10 ps, and the delay range is consistent with the design specifications. As shown in Figure 19b, although the delay accuracy of the entire frequency band meets the design requirements, the delay error in the low-frequency part is still relatively large. Therefore, consider releasing the lowest two masked bits and correcting states with large errors to improve the delay accuracy while meeting the design specifications. The delay correction method is shown in Figure 20.

Figure 20a shows the compensation situation when the actual delay is less than the ideal delay and the difference is large. Taking the ideal delay of 630 ps as an example, if the measured delay is 622 ps and the lowest two bits are released for compensation, the originally blocked lowest two bits will change from $\left[00\right]$ to $\left[11\right]$, theoretically compensating for 7.5 ps. The measured compensation amount is 7 ps, and the compensated delay is 622+7=629 ps.

Taking the ideal delay of 630 ps as an example, if the measured delay is 635 ps, the above compensation scheme cannot be used. At this point, selecting the previous ideal delay state of 620 ps resulted in a measured delay of 626 ps. Using this state and releasing the lowest two bit compensation, the lowest two bits change from $\left[00\right]$ to $\left[11\right]$, theoretically compensating for 5 ps. The measured compensation amount is 4.5 ps, and the delay after compensation is 626+4.5=630.5 ps.

### 4.3. The Impact of Delay Correction on Beam Pointing

**Figure 21:**
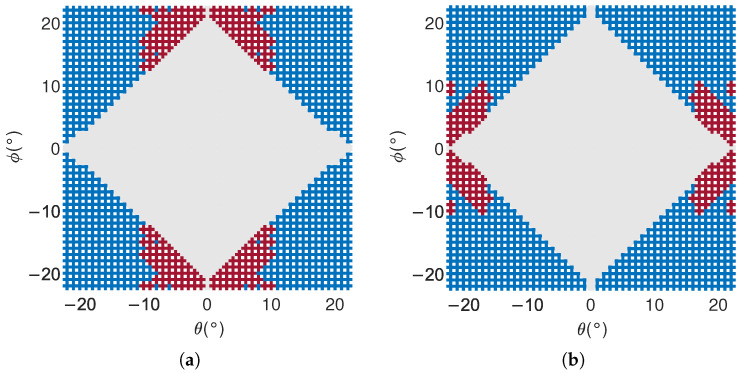
Comparison of beam pointing accuracy before and after correction: (**a**) accuracy of angle measurement in θ direction; (**b**) accuracy of angle measurement in ϕ direction.

For 18 GHz phased array RF systems, beam scanning is achieved through a combination of delay modules and phase-shifting modules. In an 8×8 phased array RF system with sixty-four antenna elements, each antenna element is connected to an independent phase shifter. Sixty-four signals are combined into four signals through a two-stage power merging link, corresponding to the four antenna subarrays mentioned in Section 3.3. These four signals are input into four independent delay channels of the delay module to achieve delay function. When scanning with the phased array, the delay module first delays the four antenna subarrays separately to achieve path differences in the signals between subarrays. Then, the phase shifter is used to achieve the signal path difference between the 16 antenna units in the subarray. Therefore, the delay accuracy of the delay device is the primary factor affecting beam pointing. For the delay module FE04, the original measurement data and the delay-corrected measurement data are used for simulation to obtain the corresponding delay accuracy simulation results. The corresponding images are shown in Figure 21.

The beam pointing of the system was simulated using a simulation model, with a simulation range of $\theta =-22^{\circ }$:$1^{\circ }$:$22^{\circ }$, $\phi =-22^{\circ }$:$1^{\circ }$:$22^{\circ }$, a total of 2025 simulation points. As shown in Figure 21, the gray area indicates that the beam pointing accuracy remains unchanged before and after FE04 correction. This is because, during small-angle scanning, the delay parameter of FE04 is small and the delay error is also small. Therefore, the delay parameter before and after FE04 correction has not changed, and the beam pointing accuracy has not changed either.

The blue area represents the improvement in beam pointing accuracy after FE04 correction. This is because, during large-angle scanning, the delay parameter of FE04 is relatively large, and the delay error is also large. After FE04 correction, the delay accuracy is improved, and the beam pointing accuracy is also improved accordingly.

However, the beam pointing accuracy is also related to the phase shifter. In the simulation, the phase shifter adopts 7-bit quantization, with a minimum phase shift of $2.8125^{\circ }$. When the required phase shift is less than $2.8125^{\circ }$, the antenna array elements cannot reach the set scanning direction. When the delay and phase shifter have opposite effects on beam pointing, the beam pointing accuracy will actually be improved. Therefore, for the red area in Figure 21, although the delay accuracy has been improved after FE04 correction, the beam pointing accuracy has decreased.

Overall, the beam pointing errors of 2025 simulated points were statistically analyzed, and their corresponding root mean square errors were compared after delay correction in FE04. The results are shown in Table 4:

From the beam pointing error data of the five selected frequency bands in Table 4, it can be seen that, except for 2 GHz, the errors in the θ and ϕ angles of the beam pointing in the other four frequency bands are significantly reduced after delay correction. This demonstrates the effectiveness of the delay control strategy correction. For 2 GHz, the beam pointing error before and after delay correction did not change. This is because the scanning angle corresponding to the beam scanning area is small, and the required delay is within 100 ps. Within this delay range, the original delay parameter error corresponding to 2 GHz is very small, so no correction has been made.

Similarly, within this delay range, as the frequency increases, the delay error gradually increases. Therefore, as shown in Table 4, the improvement in beam pointing error after delay correction also increases with the increase in frequency.

## 5. Conclusions

This article builds a broadband signal simulation model of a phased array RF system based on the broadband phased array RF system being developed by Nanhu Laboratory. Compared with the existing behavioral-level simulation tools, such as Matlab’s Phased Array System Toolbox and AWR, the simulation model proposed in this paper has the following characteristics: it can achieve the full process simulation analysis from the phased array antenna to the RF link in one step; behavior-based simulation models can accelerate simulation speed, simplify simulation links, and improve system design and optimization efficiency; and the simulation model supports broadband signal simulation and can accurately estimate and evaluate the functional indicators of the system (such as beam scanning and angle measurement accuracy), providing a scientific basis for the system design. More importantly, it reserves measured parameter interfaces for each module of the phased array, which has broad prospects in terms of the authenticity of simulation results.

At present, the behavior-level signal simulation model of the established broadband RF system has been encapsulated into independent simulation software. A preliminary simulation analysis was conducted on the broadband RF system RFS04 under development at Nanhu Laboratory using self-developed simulation software. We built a simulation model of the RF system based on the calculation principle shown in Section 2. The calculation results of relevant parameters are shown in Section 3. The results show that the antenna pattern, beam pointing accuracy, and angle measurement accuracy of the system all meet the design requirements, and increasing the quantization bits of the phase shifter and delay can improve the beam pointing accuracy. This article takes the scanning range of $\theta =\left[0^{\circ },60^{\circ }\right]$ and $\phi =\left[-45^{\circ },45^{\circ }\right]$ as an example to explore the beam scanning strategy under the condition of the optimal array tilt angle of 22.21°. After simulation analysis, the corresponding 3 dB beam coverage rate is 91.9%, and the beam utilization rate is 68.7%. The parameter indicators of this arrangement are closer to those of the staggered arrangement, meeting the requirement of a beam coverage rate of over 85% in the design. This article also used the sum difference amplitude angle measurement method to simulate and analyze the angle measurement accuracy of the system, verifying that the angle measurement accuracy improves with an increase in the signal-to-noise ratio. Therefore, in the design of phased array RF systems, the signal-to-noise ratio of the output signal should be maximized. Finally, a simulation model was used to analyze the delay module of the phased array system under development. The simulation results showed that, after correcting the delay parameters of FE04, the beam scanning accuracy was improved, proving the effectiveness of the delay correction method. This also proves that improving the quantization accuracy of delay devices can enhance the beam pointing accuracy of phased arrays. These simulation results validate the correctness and practicality of the simulation model.

Although the modeling work of the broadband phased array system has been essentially completed, further refinement is still required. Firstly, it is essential to conduct parameter measurements for a broader range of hardware modules and integrate them into a digital twin module library. This will support the simulation of a wider variety of phased array RF systems. Secondly, given that the system employs signal simulation techniques, there is still a need to enhance the simulation speed. This calls for continuous algorithm optimization and the support of more powerful computers.

## Figures and Tables

**Table 1:** The influence of quantization bit number of phase shifter on beam pointing.

Frequency	3 bits	4 bits	5 bits	6 bits	7 bits	8 bits	9 bits	10 bits
2 GHz	14.87	11.66	8.31	6.30	5.45	4.53	3.92	3.35
10 GHz	2.38	1.59	1.60	1.52	1.16	1.03	0.86	0.81
18 GHz	1.72	1.23	0.88	0.85	0.62	0.61	0.50	0.44

**Table 2:** Beam scanning angles.

θ	$\phi_{1}$	$\phi_{2}$	$\phi_{3}$	$\phi_{4}$	$\phi_{5}$	$\phi_{6}$	$\phi_{7}$
$0^{\circ }$	$-45^{\circ }$	$-30^{\circ }$	$-15^{\circ }$	$0^{\circ }$	$15^{\circ }$	$30^{\circ }$	$45^{\circ }$
$15^{\circ }$	$-45^{\circ }$	$-27^{\circ }$	$-9^{\circ }$	∖	$9^{\circ }$	$27^{\circ }$	$45^{\circ }$
$30^{\circ }$	∖	$-45^{\circ }$	$-22.5^{\circ }$	$0^{\circ }$	$22.5^{\circ }$	$45^{\circ }$	∖
$45^{\circ }$	∖	$-45^{\circ }$	$-15^{\circ }$	∖	$15^{\circ }$	$45^{\circ }$	∖
$60^{\circ }$	∖	∖	$-45^{\circ }$	$0^{\circ }$	$45^{\circ }$	∖	∖

**Table 3:** Delay device parameters.

Parameters	Symbol	Design Indicators
Frequency	*f*	2~18 GHz
Delay step	$T_{step}$	10 ps
Bits of delay device	$k_{bit}$	6 bit
Delay range	$T_{range}$	0~630 ps
Receiving delay accuracy	$RMS_{R}$	<5 ps
Uneven gain within 1 GHz range for receiving	$\Delta G_{R}$	<1 dB
Transmission delay accuracy	$RMS_{T}$	<5 ps
Uneven gain within 1 GHz range of transmission	$\Delta G_{T}$	<1 dB

**Table 4:** Comparison of beam pointing error before and after delay correction.

Frequency	$\Delta \theta_{0}$	$\Delta \theta_{m}$	$\Delta_{\Delta \theta }$	$\Delta \phi_{0}$	$\Delta \phi_{m}$	$\Delta_{\Delta \phi }$
2 GHz	$0.2636^{\circ }$	$0.2636^{\circ }$	$0^{\circ }$	$0.2734^{\circ }$	$0.2734^{\circ }$	$0^{\circ }$
6 GHz	$0.3159^{\circ }$	$0.3063^{\circ }$	$0.0096^{\circ }$	$0.3184^{\circ }$	$0.3111^{\circ }$	$0.0073^{\circ }$
10 GHz	$0.2448^{\circ }$	$0.2340^{\circ }$	$0.0108^{\circ }$	$0.2577^{\circ }$	$0.2462^{\circ }$	$0.0115^{\circ }$
14 GHz	$0.3207^{\circ }$	$0.2550^{\circ }$	$0.0657^{\circ }$	$0.3380^{\circ }$	$0.2699^{\circ }$	$0.0681^{\circ }$
18 GHz	$0.5176^{\circ }$	$0.2865^{\circ }$	$0.2311^{\circ }$	$0.5321^{\circ }$	$0.2848^{\circ }$	$0.2473^{\circ }$

## Data Availability

Data are contained within the article.
